# Clinical characteristics and treatment outcomes of germ cell tumor in Jordan: A tertiary center experience

**DOI:** 10.1080/2090598X.2022.2163473

**Published:** 2023-01-03

**Authors:** A. El-Achkar, H. Alasadi, J. El-Asmar, A. Armache, R. Abu-Hijlih, F. Abu-Hijle, A. Al-Ibraheem, J. Khzouz, S. Salah, M. Shahait

**Affiliations:** a Department of Surgery, Division of Urology, American University of Beirut, Beirut, Lebanon; b Department of Surgery, King Hussein Cancer Center, Amman, Jordan

**Keywords:** Testicular cancer, germ cell tumor, testicular tumor, RPLND

## Abstract

**Objective:**

In the Middle East, there is a paucity of data regarding germ cell tumor characteristics and treatment outcomes. Herein, we aim to present the largest series in Jordan reporting our cancer center experience managing GCT.

**Methods:**

Between 2010 and 2020, a total of 241 patients with a pathological diagnosis of GCT were treated at our cancer center. Demographic, epidemiologic, and pathological data were retrospectively collected. In addition, survival and relapse outcomes based on tumor stage and adjuvant treatment were collected.

**Results:**

A total of 241 patients were diagnosed with GCT, of whom 108 (44.8%) had seminoma and 133 (55.2%) had non-seminoma tumors (NSGCT). Median age (interquartile range) at diagnosis was 31 years (25–36). Patients with seminoma (68.5%) had pT1 disease post orchiectomy, while only 37.5% of patients with NSGCT had pT1 on final pathology. Elevated tumor markers such as beta-human chorionic gonadotropin were present in 10% of seminomas. Following radical orchiectomy and staging, 88 (36.5%) went for active surveillance while 153 patients (63.5%) received adjuvant treatment. With regard to pathology slides read outside, rereading by our genitourinary pathologist yielded a discrepancy on GCT type in 41 (19.3%) out of 212 patients. The median follow-up was 36 (24–48) months. Twenty-two patients relapsed after an average follow-up time of 39 months. The 5-year overall survival for stage I, II, and III was 98%, 94%, and 87%, respectively, and 3-year recurrence-free survival for stage I, II, and III was 94.8%, 78%, and 67%, respectively.

**Conclusion:**

Our data on testicular GCT including demographic, histological, and treatment outcomes were comparable to that of developed countries. In light of the pathology discrepancy rate revealed in our study, authors recommend a second review by expert genitourinary pathologists to ensure proper classification and management of GCT.

## Introduction

Testicular cancer is the 25th most commonly diagnosed cancer in North America, and it accounts for 1–3% of all male cancers in Europe and globally [1]. To date, it is the most common cancer among young men, with half of the cases being diagnosed between the ages of 20 and 34 [2]. Worldwide, a 44-fold increased incidence of testicular cancer was recorded in 2012 where the Middle East witnessed a notable increase of 2–4% in the incidence of testicular cancer [1]. Nevertheless, advances in treatment modalities maintained a relative survival rate as high as 95.3% worldwide [3,4]. In the Arab world and Egypt specifically, mortality over incidence of testicular cancer is 17.6%, which is significantly better than all reported genitourinary cancers (46.7–57%), which a reflection of the excellent survivorship of testicular cancer in Egypt and the rest of world [5–7].

Currently, there is a paucity of epidemiologic data regarding testicular cancer in the Middle East [8]. A notable study from Saudi Arabia revealed a substantial increase in testicular cancer incidence among the young Saudi population [9], while another study from Lebanon showed a comparable distribution of testicular germ cell tumors (GCTs) with developed countries [10]. Similarly, treatment outcomes from another Lebanese cohort was comparable to other countries worldwide [11].

Hence, we sought to present the largest Middle Eastern series to date that reports on the histopathologic distribution of GCTs as well as the demographics and treatment outcomes of patients with GCTs.

## Methods

This is a single-center retrospective study on patients with histologically proven GCT who were treated at the King Hussein Cancer Center (KHCC). This study was granted ethical community approval and an Institutional Review Board approval. Consents were waived. The data were obtained from the KHCC charts, which included all patients with testicular GCT who were treated at the institution between the years 2010 and 2020. Patient demographics, clinical, and pathological data were retrospectively collected by chart review. Patients underwent radical orchiectomy for primary staging either at KHCC or at an outside institution. As part of the cancer center protocol, all pathological specimens done outside were secondarily reviewed by a specialized genitourinary pathologist prior to initiation any treatment. Clinical staging was based on the 2018 *AJCC Cancer Staging Manual* [12]. Relevant data on tumor markers and follow-up treatments including chemotherapy regimens, retroperitoneal lymph node dissection (RPLND), or radiation therapy were also collected. Data on survival were retrieved from the country cancer registry, which has up-to-date information on survival of patients even if they were lost to follow-up. The cancer registry in Jordan is linked to the Ministry of Interior, and death reports are updated daily.

## Statistical analysis

Continuous variables were described with mean and interquartile range (IQR), whereas categorical variables were described with count and percentage. For normally distributed continuous variables, independent-*T* test was used to test for significance, while for categorical variables, Chi-squared test was used to test for significant association between an independent (predictor) and dependent (outcome) variables. Two-sided statistical significance was set at *P*-value <0.05 for all variables. All analyses were conducted using the statistical analysis software platform (IBM SPSS) For MAC version 28.0.0.0 (SPSS, Chicago, IL, USA).

## Results

### Patient and tumor characteristics

A total of 241 patients diagnosed with GCT of the testis were included in this study with a median (IQR) age at diagnosis of 31 years (25–36). Forty-seven percent of the patients had tumors on the right, while 52% had tumors on the left, whereas synchronous bilateral involvement was seen in less than 1% of cases. (Table 1) Moreover, 210 (87%) patients diagnosed with GCT were <40 years of age, while only 31 (13%) were ≥40 years of age (Table 2).

**Table 1.** Clinical characteristics of patients with testicular germ cell tumors.

	*N*	%	Median (IQR)
All GCT	241	100%	31 (25–36)
Seminoma	108	44.8%	32 (28–38)
pTx	6	5.6%	37.5 (28–40)
pT1	74	68.5%	32.5 (29–37)
pT2	26	24.1%	30.5 (25–39)
pT3	2	1.9%	35
Non-seminoma	133	55.2%	30 (25–34.5)
pTx	8	6%	31.5 (24–36)
pT1	50	37.5%	29 (25–34)
pT2	64	48.1%	30 (25–34)
pT3	10	7.5%	36 (23–36.5)
pT4	1	0.8%	29
Stages of GCT	241	100%	31 (25–36)
Stage I–III	234	97%	31 (25–36)
Unknown stage	7	3%	30 (28–39)
Stage I	130	54%	31 (26–36)
Ia	79	33%	32 (28–37)
Ib	41	17%	28 (24–33)
IS	10	4%	31.5 (26–37)
Stage II	59	24.5%	32 (26–38)
IIA	27	11.2%	31 (26–36)
IIB	15	6.2%	29 (26–34)
IIC	17	7.1%	34 (28–41)
Stage III	45	18.7%	31 (23–36)
IIIx	15	6.2%	31 (23–34)
IIIA	11	4.6%	32 (28–34)
IIIB	1	0.4%	36
IIIC	18	7.5%	28 (21–36)
Laterality			
Right	82	34%	31 (25–36)
Left	91	37.8%	31 (26–36)
Bilateral synchronous	1	0.4%	25
Unknown	67	27.8%	31 (26–37)

GCT, germ cell tumor; CS, clinical stages; IQR, interquartile range; pT, pathological; NS, nonseminoma. IIIx = stage III (not subclassified as A, B, or C)

**Table 2.** Comparison of age groups <40 and ≥40 years.

	<40	≥40	*P*-value
All GCT	210 (87%)	31 (13%)	
Seminoma	89 (82%)	19 (18%)	0.048
Non-seminoma	121 (91%)	12 (9%)	
Pathological stage (all)	148	17	
pT1	107 (53.8%)	17 (60.7%)	<0.0001
>pT1	92 (46.2%)	11 (35.7%)	
Stage I	117 (57.4%)	13 (43%)	0.15
Stage II and III	87 (42.6%)	17 (57%)	
Seminoma			
pT1	63 (74%)	11 (64.7%)	0.42
>pT1	22 (26%)	6 (35.3%)	
Stage I	65 (76%)	11 (61%)	
Stage II	17 (19%)	4 (22%)	
Stage III	4 (5%)	3 (17%)	
Non-seminoma			
pT1	44 (38.6%)	6 (54.5%)	0.30
>pT1	70 (61.4%)	5 (45.5%)	
Stage I	52 (44%)	2 (17%)	
Stage II	31 (26%)	7 (59%)	
Stage III	35 (30%)	3 (24%)	

Horizontal boxes denote relative proportions (%) of clinical characteristics in age groups <40 and ≥40 years.

A total of 108 (44.8%) patients had seminoma, while 133 (55.2%) had non-seminoma tumors (NSGCT). The median age (IQR) for the seminoma group was 32 years (28–38), while the latter had a median age (IQR) of 30 years (25–34.5) (Table 1). The majority of patients with seminoma (68.5%) had pT1 disease post radical orchiectomy, while only 37.5% of patients with NSGCT had pT1 on final pathology. Elevated tumor markers such as beta-human chorionic gonadotropin were present in 10% of seminomas. To note, the NSGCT group had a higher percentage of initial presentation with elevated markers (Table 3).

**Table 3.** Comparison of seminoma with non-seminoma, excluding pTx.

Parameter	Seminoma (*n* = 108)	Non-seminoma (*n* = 133)	*P*-value
Average age	32	30	*P* = 0.003
Proportion of pT1 n (%)	74 (68.5%)	50 (37.5%)	*P* < 0.0001
Positive serum markers	11 (10%)	32 (24%)	*P* = 0.006
Elevated β-HCG n (%) *Normal range <5 IU/I*	8 (7.4%)	23 (17.2%)	*P* = 0.032
Elevated AFP n (%) *Normal Range <40 µg/I*	0	17 (12.8%)	*P* < 0.0001

IQR, interquartile range; CS, clinical stages; pT, pathological; β-HCG, beta-human chorionic gonadotropin; AFP, alpha-fetoprotein; LDH, lactate dehydrogenase

### Histopathology and second specialized pathology review

Of the 212 slides reread by a genitourinary pathologist at KHCC, a discrepancy rate of 19.8% was found. Forty-one patients had different pathology classification upon rereading. A total of 14 (6.6%) patients with seminoma were reclassified as NSGCT, and 7 (3.3%) NSGCT were reclassified as seminoma. In addition, seven patients with mixed GCT were reclassified as embryonal (5) and teratoma (2) subtypes (Table 4).

**Table 4.** Congruence and discrepancy between the pathology slides read outside and the ones reread at KHCC, by an expert GU genitourinary pathologist.

Type of histology	Original histology (*N* = 241)	Reread histology (*N* = 212)
Seminoma	112 (46.4%)	92 (43.4%)
Non-seminoma	121 (50%)	120 (56.6%)
Other	8 (3.3%)	0
	Discrepancy rate [*N* = 41 (19.3%)] of the patients	
Seminoma → To NSGCT	14 (6.6%)	
NSGCT → To seminoma	7 (3.3%)	
NSGCT → NSGCT	*N* = 12 (5.6%)	
(Mixed → Embryonal)	5	
(Mixed → Teratoma)	2	
(Embryonal → Mixed)	3	
(Yolk → Mixed)	1	
(Teratoma → Mixed)	1	
Other → Seminoma or NSGCT	*N* = 8 (3.7%)	
(Intratubular germ cell → Seminoma)	1	
(Germinoma → Seminoma)	2	
(Germinoma → Mixed)	4	
(Germinoma → Embryonal)	1	

### Treatment and outcomes

**Figure 1.**
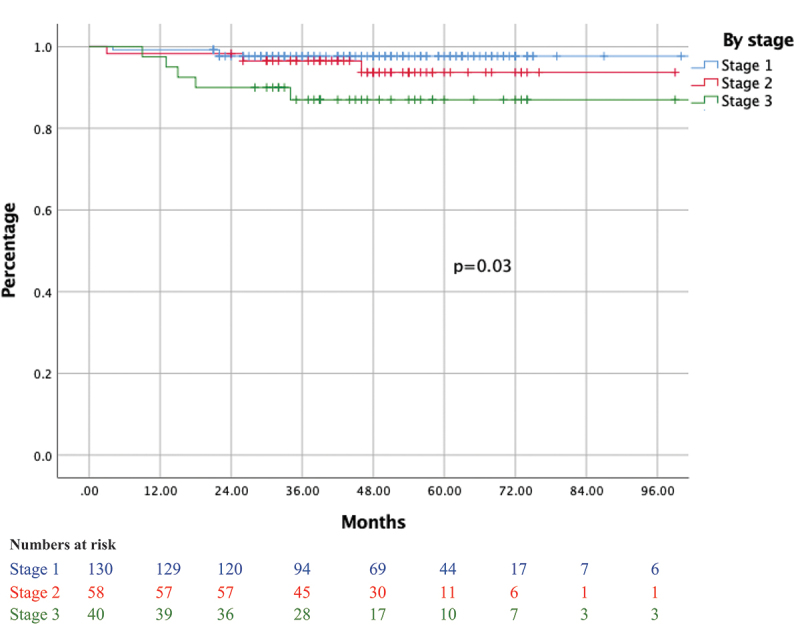
Overall survival of patients with GCT by stage.

**Figure 2.**
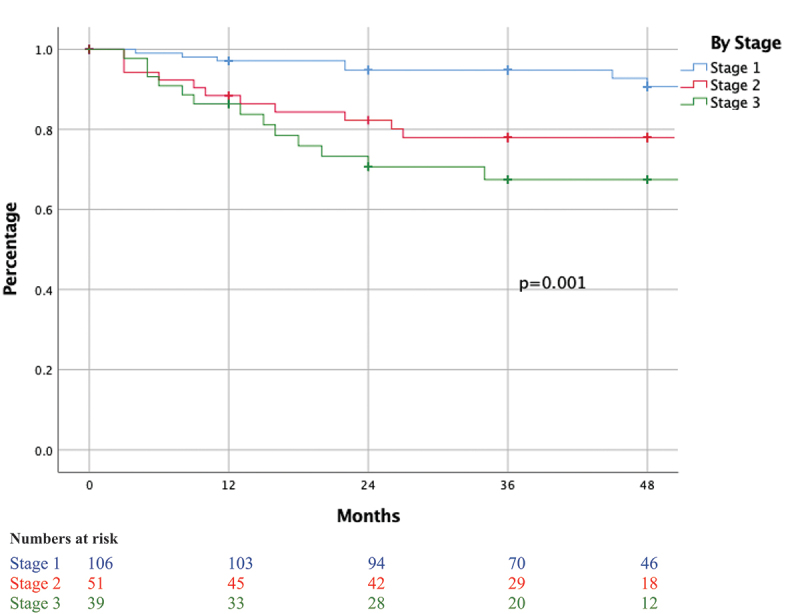
Recurrence-free survival of GCT tumor patients by stage.

Following radical orchiectomy and staging, 153 (63.4%) patients received adjuvant treatment, whereas 88 (36.5%) patients underwent active surveillance. Among the former group, chemotherapy was the most common type of adjuvant therapy administered with the majority receiving a bleomycin, etoposide, and platinum combination (108/125). Adjuvant radiotherapy was administered to 22 (9.1%) patients, and adjuvant chemoradiation was administered to 6 (2.5%) patients (Table 5). The median (IQR) follow-up was 36 (24–48) months where 22 patients relapsed after an average follow-up time of 39 months. In contrast, 173 patients did not recur over a 5-year period while 46 (19%) patients were lost to follow-up. In addition, 6 patients with seminoma and 19 with NSGCT underwent salvage RPLND (Table 6). Five-year overall survival (OS) for stages I, II, and III is 98%, 94%, and 87%, respectively, and 3-year recurrence-free survival (RFS) for stages I, II, and III is 94.8%, 78%, and 67%, respectively (Figures 1 and 2).

**Table 5.** Management post radical orchiectomy, active surveillance, types of adjuvants treatments, and recurrence.

Adjuvant course	
Surveillance	88 (36.5%)
Radiation	22 (9.1%)
Chemotherapy	125 (52%)
BEP	108
BEP + other	8
TIP or VIP	4
Other	5
Chemo radiation	6 (2.5%)
BEP	4
Other	2
Recurrence	22
No recurrence	173
Loss to follow-up	46 (19%)

**Table 6.** Adjuvant treatment, recurrence, and survival of testicular germ cell tumors divided by stages and histology.

	Histology									
	Seminoma	Non-seminoma	Ia	Ib	Is	IIa	IIb	IIc	III (IIIa-IIIc)	Unknown stage
Seminoma			56 (53.8%)	18 (17.3%)	2 (1.9%)	9 (8.7%)	4 (3.8%)	8 (7.7%)	7 (6.7%)	4
Non-seminoma			23 (17.7%)	23 (17.7%)	8 (6.2%)	18 (13.8%)	11 (8.5%)	9 (6.9%)	38 (29.2%)	3
No adjuvant treatment	50 (46.3%)	38 (28.6%)	48 (60.8%)	21 (51.2%)	7 (70%)	2 (7.4%)	2 (13.3%)	2 (11.8%)	2 (4.4%)	4 (57.1%)
Chemotherapy	32 (29.6%)	93 (69.9%)	18 (22.8%)	15 (36.6%)	3 (30%)	22 (81.5%)	10 (66.7%)	14 (82.4%)	42 (93.3%)	1 (14.3%)
Radiation	22 (20.4%)	0	12 (15.2%)	5 (12.2%)	0	2 (7.4%)	2 (13.3%)	0	0	1 (14.3%)
Chemo radiation	4 (3.7%)	2 (1.5%)	1 (1.3%)	0	0	1 (3.7%)	1 (6.7%)	1 (5.9%)	1 (2.2%)	1 (14.3%)
RPLND	6 (5.6%)	19 (14.3%)								
Death	3 (2.8%)	8 (6%)								
Survival	105 (97.2%)	125 (94%)								

## Discussion

Testicular GCT represents a heterogeneous group of neoplasms with different pathologies, incidences, prognosis, and management. While the demographics of GCT and treatment outcomes are well studied in developed countries, there are minimal data about GCT in the Middle East. There are few retrospective cohorts with a limited sample population from Lebanon, Egypt, Turkey, and Saudi Arabia [9–11,13–16]. We herein present the largest single-center series in the Middle East, presenting epidemiologic and treatment outcomes of a total of 241 patients with GCT of which 108 (44.8%) were diagnosed with seminoma and 133 (55.2%) with NSGCT.

Data from western developed countries revealed that the majority of GCT are seminoma tumors (range 50–57%) [17–20]. Similarly, Surveilance, Epidimiology and End Results (SEER) data also show a higher percentage of seminoma to NSGCT [10,15], whereas our series had a higher percentage of NSGCT (55.2%) compared to seminoma (44.8%). This was also different to the already published literature from the Middle Eastern region where data from Lebanon and Saudi Arabia revealed a higher percentage of seminoma compared to non-seminoma. One plausible explanation lies in the nature of our cohort that fails to capture the incidence of testicular cancer on a national level but rather limits its registry to those patients that have been referred to our tertiary referral cancer center; hence, a biased referral of the more complex NSGCT cases could have swayed the incidence rate in favor of NSGCT. Interestingly, data from other referral centers in under-developed countries such as Pakistan and Morocco revealed similar higher rates of NSGCT [21,22]. The median age (IQR) for all GCT was 31 (25–36) years. For seminoma, the median age (IQR) at diagnosis was 32 (28–38) years, while the median age (IQR) for NSGCT was 30 (25–34.5) years. While both groups reveal a high incidence of diagnosis in young adults, NSGCT patients presented earlier with an average age of 32 compared to 30, *P* = 0.003 (Table 2). The age of presentation of GCT patients along with the presentation age of the two different subtypes was similar to the already published data from around the globe [11,23]. Nevertheless, several cohorts report various ranges of age presentation specially with NSGCT. For instance, the mean age of NSGCT in Pakistan and the US was reported to be 23 and 25 years, respectively, whereas in Germany, it was 31 years [21,23].

It is without doubt that for GCT patients, age at presentation is of utmost importance when it comes to survival rates. The sooner the age of presentation, the higher chances of survival [7,24]. In fact, testicular cancer mortality doubles in those diagnosed at age 40+ [24]. In our data, 18% of seminoma patients initially presented at 40 years of age or above, whereas less than 9% of patients with NSGCT initially presented at 40 years of age or above (Table 3).

High levels of awareness play a major role in early detection of testicular tumor [25]. Reports in the region reveal that the majority of GCT patients present for treatment early in their disease with the majority revealing that most initially present with localized disease [11,15,16]. Compared with seminoma patients, a higher percentage of patients with NSGCT are diagnosed with regional or distant disease on presentation. Our cohort revealed that 17% of seminoma patients presenting at age 40 or above had metastatic disease, whereas 24% of NSGCT aged 40 or older had metastatic disease. This further strengthens the association between age of presentation and OS.

Our cohort demonstrated a slightly higher incidence of left-sided testicular involvement (52%). As cryptorchidism is more common on the right side, several studies revealed a side discrepancy favoring the development of testicular cancer on the right side [10, 26]. Such discrepancy could be explained by our relatively small sample size. Furthermore, the rate of bilateral testicular cancer in our series was low encompassing to 0.4% of patients. This was in line with previously published series revealing a rate as low as 0.6% [27,28].

Our institutional protocol mandates a confirmatory pathology review by an experienced genitourinary pathologist if orchiectomy was done outside, prior to the initiation of any treatment modality. Our data revealed a 19.8% discrepancy rate between original pathology reports and those reread by the specialized pathologist. Fifteen (7%) patients who were initially read as seminoma were reread as NSGCT, while seven patients who were initially read as NSGCT were reread as seminoma. In addition, seven patients read as mixed were reread as embryonal and teratoma tumors. Nason et al. reported that among specimens from the Ontario Cancer Registry who underwent a second pathology review, 40% yielded significant changes in pathological parameters such as T stage and lymphovascular invasion (LVI), whereas the rate of histopathological subtype change was 5.4% [29]. Another study by Harari et al. in 2017 showed a histopathological discrepancy rate of 31% [30]. These significant levels of histopathological discrepancies strengthen the role of a secondary confirmatory review by genitourinary specialist pathologist, especially that treatment strategies in testicular cancer are highly influenced by stage of disease and pathological subtype.

GCT management protocols involve post-surgical staging upon which adjuvant treatment versus rigorous active surveillance is decided. In our cohort, 64% of patients received adjuvant treatment including chemotherapy (52%), radiation (9.1%), and chemoradiation (2.5%), with 5-year OS of 97% (Figure 1). In other countries such as Spain, adjuvant chemotherapy is at an almost similar rate of 50% [31]. For stages Ia and Ib, 39.2% and 48.8% of patients underwent adjuvant treatment, respectively. For those with stage I seminoma, 43% underwent adjuvant treatment with a recurrence rate of 2.6%, whereas those with stage I non-seminoma, 42% underwent adjuvant chemotherapy with a recurrence rate of 4.7%. Compared to other series, ours revealed a slightly higher percentage of stage I patients receiving adjuvant treatment, and lower recurrence rate. In other series, this increased tendency for adjuvant chemotherapy in stage I is prevalent in third world countries where there is significant cost for meticulous follow-up and low compliance in active surveillance [11,32]. Another reason might be the higher presence of LVI in our sample population which we did not account for in our series.

This study has several limitations. The retrospective nature of the study limits short interval follow-up. Although this is the largest series in the Middle East, the sample size is still small compared to some of the larger series in North America and Europe. In addition, our loss to follow-up rate is relatively high at 19%; nevertheless, we believe this study is noteworthy with good inputs on treatment of GCT in the Middle East; however, this region needs larger multicenter studies to further understand the nuances of treatment of GCTs according to stage, especially in areas with limited access to adjuvant treatment.

Our histopathologic, demographic, and survival data are commensurate with those from around the world. This reinforces the fact that GCT remains a curable solid tumor disease of the young with excellent survival rates if diagnosed promptly and treated accordingly. As such, it is of utmost importance that genitourinary pathologists, when available, confirm subtype classification of testicular tumors prior to initiation of treatment modalities.

## Data Availability

All the data generated or analyzed during this study are not publically avaialbe; however, they are availabe upon editor / reviewer request.

## References

[1] Gurney JK, Florio AA, Znaor A, et al. International trends in the incidence of testicular cancer: lessons from 35 years and 41 countries. Eur Urol. 2019;76(5):615–623. 31324498 10.1016/j.eururo.2019.07.002 PMC8653517

[2] Chia VM, Quraishi SM, Devesa SS, et al. International trends in the incidence of testicular cancer, 1973–2002. Cancer Epidemiol Biomarkers Prev. 2010;19(5):1151–1159. 20447912 10.1158/1055-9965.EPI-10-0031 PMC2867073

[3] Coleman MP, Gatta G, Verdecchia A, et al. EUROCARE-3 summary: cancer survival in Europe at the end of the 20th century. Ann Oncol. 2003;14(5):v128–49. 14684503 10.1093/annonc/mdg756

[4] Hanna NH, Einhorn LH. Testicular cancer–discoveries and updates. N Engl J Med. 2014;371(21):2005–2016. 25409373 10.1056/NEJMra1407550

[5] Sung H, Ferlay J, Siegel RL, et al. Global Cancer Statistics 2020: GLOBOCAN estimates of incidence and mortality worldwide for 36 cancers in 185 countries. CA Cancer J Clin. 2021;71(3):209–249. 33538338 10.3322/caac.21660

[6] Elgamal A. COVID-19 pandemic, disease burden & management towards achieving sustainable development goals (SDGs) of Egypt-Vision-2030. J Instdgpt. 2020;94:207–247.

[7] Noureldin YA, Alqirnas MQ, Aljarallah MF, et al. Testicular cancer among Saudi adults: hands on a nationwide Cancer Registry over 10 years. Arab J Urol. 2022;20(1):1–7. 36353476 10.1080/2090598X.2022.2084902 PMC9639499

[8] Park JS, Kim J, Elghiaty A, et al. Recent global trends in testicular cancer incidence and mortality. Medicine (Baltimore). 2018;97(37):e12390. 30213007 10.1097/MD.0000000000012390 PMC6155960

[9] Abomelha M. Adult testicular cancer: two decades of Saudi national data. Urol Ann. 2017 Oct-Dec;9(4):305–309. 29118528 10.4103/UA.UA_11_17 PMC5656951

[10] Assi T, Rassy M, Nassereddine H, et al. Distribution of testicular tumors in Lebanon: a single institution overview. Asian Pac J Cancer Prev. 2015;16(8):3443–3446. 25921159 10.7314/apjcp.2015.16.8.3443

[11] Assi T, Nasr F, Rassy EE, et al. Characteristics of incident testicular cancer in Lebanon – 1990–2015 single institutional experience. Asian Pac J Cancer Prev. 2016;17(4):1899–1902. 27221873 10.7314/apjcp.2016.17.4.1899

[12] Amin M, Edge S, Greene F. AJCC Cancer Staging Manual. 8th ed. New York City: Springer Cham; 2017.

[13] Zawam HH, Selim A, Osman NO, et al. Factors influencing the response rate and survival of testicular germ cell tumors: a single institution experience from Egypt. Res Oncol. 2021;17(2): 66–72.

[14] Gumus M, Bilici A, Odabas H, et al. Outcomes of surveillance versus adjuvant chemotherapy for patients with stage IA and IB nonseminomatous testicular germ cell tumors. World J Urol. 2017;35(7):1103–1110. 27812752 10.1007/s00345-016-1964-6

[15] El-Hsseiny G. Germ cell tumors in undescended testis-prognostic factors and treatment outcome. J Egypt Natl Canc Inst. 2001;13(3):209–214.

[16] Ozgun A, Karagoz B, Tuncel T, et al. Clinicopathological features and survival of young Turkish patients with testicular germ cell tumors. Asian Pac J Cancer Prev. 2013;14(11):6889–6892. 24377621 10.7314/apjcp.2013.14.11.6889

[17] Trabert B, Chen J, Devesa SS, et al. International patterns and trends in testicular cancer incidence, overall and by histologic subtype, 1973–2007. Andrology. 2015;3(1):4–12. 25331326 10.1111/andr.293 PMC4410839

[18] Sant M, Aareleid T, Artioli ME, et al. Ten-year survival and risk of relapse for testicular cancer: a EUROCARE high resolution study. Eur J Cancer. 2007;43(3):585–592. 17222545 10.1016/j.ejca.2006.11.006

[19] Richiardi L, Bellocco R, Adami HO, et al. Testicular cancer incidence in eight Northern European countries: secular and recent trends. Cancer Epidemiol Biomarkers Prev. 2004;13(12):2157–2166. 15598775

[20] Ruf CG, Isbarn H, Wagner W, et al. Changes in epidemiologic features of testicular germ cell cancer: age at diagnosis and relative frequency of seminoma are constantly and significantly increasing. Urol Oncol. 2014;32(1):33.e1–6. 10.1016/j.urolonc.2012.12.002 23395239

[21] Mushtaq S, Jamal S, Mamoon N, et al. The pathological spectrum of malignant testicular tumours in northern Pakistan. J Pak Med Assoc. 2007 Oct;57(10):499–501. 17990425

[22] Raiss GG, Andaloussi MM, Raissouni SS, et al. Spermatocytic seminoma at the National Institute of Oncology in Morocco. BMC Res Notes. 2011;4:218. 21714871 10.1186/1756-0500-4-218 PMC3195761

[23] Stang A, Jansen L, Trabert B, et al. Survival after a diagnosis of testicular germ cell cancers in Germany and the United States, 2002–2006: a high resolution study by histology and age. Cancer Epidemiol. 2013;37(4):492–497. 23623488 10.1016/j.canep.2013.03.017 PMC4024392

[24] Fosså SD, Cvancarova M, Chen L, et al. Adverse prognostic factors for testicular cancer-specific survival: a population-based study of 27,948 patients. J Clin Oncol. 2011;29(8):963–970. 21300926 10.1200/JCO.2010.32.3204

[25] Saab M, Noureddine S, Abu-Saad Huijer H, et al. Surviving testicular cancer: the Lebanese lived experience. Nurs Res. 2014;63(3):203–210. 24785248 10.1097/NNR.0000000000000033

[26] Swerdlow AJ, Higgins CD, Pike MC. Risk of testicular cancer in cohort of boys with cryptorchidism. BMJ. 1997;314(7093):1507–1511. 9169396 10.1136/bmj.314.7093.1507 PMC2126779

[27] Miki T, Kamoi K, Fujimoto H, et al. Clinical characteristics and oncological outcomes of testicular cancer patients registered in 2005 and 2008: the first large-scale study from the Cancer Registration Committee of the Japanese Urological Association. Int J Urol. 2014;21(8):S1–6. 24725194 10.1111/iju.12441

[28] Hentrich M, Weber N, Bergsdorf T, et al. Management and outcome of bilateral testicular germ cell tumors: twenty-five year experience in Munich. Acta Oncol. 2005;44(6):529–536. 16165911 10.1080/02841860510029923

[29] Nason GJ, Sweet J, Landoni L, et al. Discrepancy in pathology reports upon second review of radical orchiectomy specimens for testicular germ cell tumors. Can Urol Assoc J. 2020;14(12):411–415. 32574142 10.5489/cuaj.6481 PMC7704081

[30] Harari SE, Sassoon DJ, Priemer DS, et al. Testicular cancer: the usage of central review for pathology diagnosis of orchiectomy specimens. J Urol Oncol. 2017 ;35(10):605.e9–605.e16. 10.1016/j.urolonc.2017.05.018 28647396

[31] Moreno A, Domínguez A, Alpuente C, et al. Clinical presentation features of testicular cancer in public hospitals in the Autonomous Community of Madrid, Spain. Actas Urol Esp. 2015;39(1):2–7. 25204991 10.1016/j.acuro.2014.03.009

[32] Amato RJ, Ro JY, Ayala AG, et al. Risk-adapted treatment for patients with clinical stage I nonseminomatous germ cell tumor of the testis. Urology. 2004 ;63(1):144–148. 14751368 10.1016/j.urology.2003.08.045

